# Prevalence and Factors Associated With Liver Fibrosis Among Adult HIV-Infected Patients Attending Urban and Rural Care Clinics in Uganda

**DOI:** 10.1093/ofid/ofaa483

**Published:** 2020-10-13

**Authors:** Clara Wekesa, Gregory D Kirk, Jim Aizire, Eve-Marie Benson, Alex Karabarinde, Rosalind Parkes-Ratanshi, Ponsiano Ocama

**Affiliations:** 1 MRC/UVRI and London School of Hygiene and Tropical Medicine Uganda Research, Entebbe, Uganda; 2 John Hopkins University, Baltimore, Maryland, USA; 3 HIV and HCC in Uganda (H2U) Consortium, Infectious Diseases Institute, Kampala, Uganda; 4 Cambridge University, Institute of Public Health, Cambridge, UK; 5 Makerere University, College of Health Sciences, Kampala, Uganda

**Keywords:** ART era, Fibroscan, HIV/AIDS, liver fibrosis, Sub-Saharan Africa

## Abstract

**Background:**

Liver fibrosis is common among HIV-infected patients. Risk factors vary by location. Understanding this variation may inform prevention strategies. We compared the prevalence and correlates of liver fibrosis among HIV-infected patients attending care clinics in Uganda.

**Methods:**

This was a cross-sectional study involving 2030 HIV-infected patients attending care clinics in urban and rural Uganda. Liver fibrosis was defined as liver stiffness measurement (LSM) >7.1 KPa. Proportions and correlates of liver fibrosis were assessed and compared using logistic regression stratified by gender and site.

**Results:**

Prevalence of liver fibrosis was higher among participants in the rural clinic (15% vs 11%; *P* = .017). History of tobacco use (urban *P* = .022; rural *P* = .035) and serologic evidence of hepatitis C infection (HCV; urban *P* = .028; rural *P* = .03) was associated with liver fibrosis in all men. Elevated liver transaminases (urban *P* = .002; rural *P* = .028) and increasing age (urban *P* = .008; rural *P* = .052) were risk factors among all women. Tobacco use among women was only a risk factor in those attending the rural clinic (*P* = .003), and detectable HIV viral load (*P* = .002) for men in the urban clinic.

**Conclusions:**

Liver fibrosis is prevalent among HIV-infected persons in Uganda. HIV viral suppression and avoiding tobacco may be strategies to prevent liver fibrosis and cancer risk.

With improved HIV/AIDS care and management, HIV-infected persons now live long enough to experience other non-HIV-related causes of disease or death, such as liver disease [1]. Liver disease is the second leading cause of death among HIV-infected persons [2]. Risk factors for liver disease have transitioned in the era of antiretroviral therapy (ART), with fewer opportunistic infections and more comorbid diseases and infections being the drivers for liver disease [3, 4]. Viral hepatitis is the major risk factor for liver disease in this population given that the routes of transmission are shared and progress to ensuing complications in comparison with the general population is faster. In the background of HIV, there is increased viral replication of hepatitis B and reduced viral shedding of hepatitis C [5]. Other risk factors include direct (infection of all types of liver cells by HIV with premature death) and indirect (cytokine-driven mechanisms stimulating increasing collagen formation) effects of chronic HIV infection, toxicity from long-term exposure to drugs including ART, exposure to aflatoxin, harmful use of alcohol, and metabolic conditions that predispose to elevated blood lipids [3, 4, 6–8]. Urbanization does influence the distribution of these risk factors geographically on a global scale and even within specific countries and consequently influences distribution of liver disease. For example, the disparity in the distribution of risk factors such as viral hepatitis, obesity, and use of alcohol and tobacco within the country has been demonstrated from various separate studies [9, 10].

Chronic inflammation of the liver resulting from any 1 risk factor develops into liver fibrosis. Liver fibrosis is the primary risk factor for complicated liver disease, notably cirrhosis and/or hepatocellular carcinoma [11]. Most studies on the etiology of liver fibrosis have been undertaken in Western populations [12]. The few studies done in Sub-Saharan Africa (SSA) were mainly conducted before the era of ART rollout, demonstrating most of the causes to be opportunistic conditions resulting from a deprived immune state [13, 14]. The few other studies done in the era of ART rollout focused on select populations, for example, hepatitis B/HIV-coinfected patients, using noninvasive methods with low accuracy for assessment of liver fibrosis [15–18]. HIV infection accelerates progression of liver fibrosis to complicated disease. Sub-Saharan Africa harbors 80% of the global burden of HIV/AIDS and would be most likely to experience an epidemic of complications of liver fibrosis if no preventive measures are taken [19, 20]. The same region has limited expertise and/or advanced therapeutic options such as liver transplant, rendering tertiary management of complications of liver fibrosis costly. Presently the region has at least a decade of experience with ART and improved longevity of people with HIV/AIDS. It would therefore be important to evaluate for other risk factors, not limited to viral hepatitis, using relatively more accurate diagnostics to understand the differences in burden and distribution of drivers for liver fibrosis in resource-limited settings, informing tailored cost-effective prevention strategies. Therefore, we undertook a study to determine and compare the prevalence and factors associated with liver fibrosis among HIV-infected patients routinely presenting to a rural primary care clinic and an urban tertiary care clinic in Uganda.

## Study Methods

This was a cross-sectional study based on pooled data from 2 different studies involving HIV-infected persons aged ≥18 years attending care clinics. The study in the rural General Population Clinic (GPC; rural clinic), a primary care clinic, involved active data collection, and the study at the urban tertiary clinic, the Adult Infectious Diseases Clinic (urban clinic) in Kampala, Uganda, was a secondary data analysis of previously collected data for a prospective study looking at the prevalence and progression of premalignant cirrhosis. Both the rural and urban clinics are well described elsewhere [21–23]. Both the rural and urban studies were conducted between January 2015 and December 2017.

## Participant Enrollment Procedure

**Figure 1.**
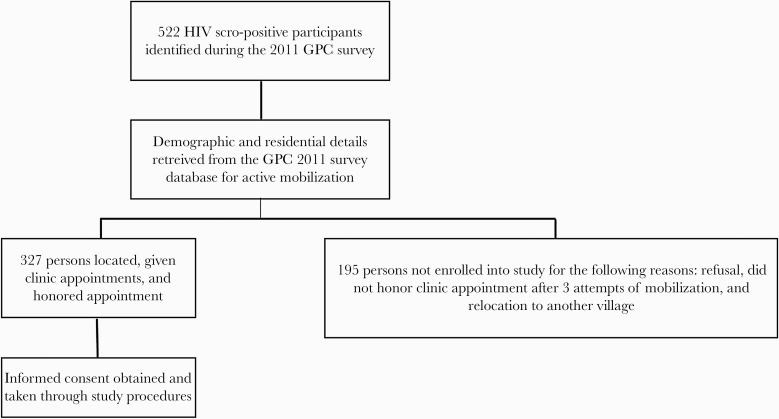
Flow chart showing the recruitment process for participants at the GPC clinic.

Participants from the rural clinic were actively mobilized from within their households in their respective villages using field research staff, having been identified as HIV sero-positive from a previous sero-survey conducted in 2011. Field research staff using residence information retrieved from the previous survey made physical visits to communicate information about the study and obtain screening consent. We sampled from all 23 villages that were within a 10-km radius of the GPC clinic. A total of 522 of 8000 (6.5%) persons were identified from the 2011 sero-survey as HIV-positive. Those who still resided within the villages and were able to be located and provided initial consent were provided an appointment date to come to the research clinic to provide informed consent for all study procedures (Figure 1). Adults with no documented or known history of liver disease and no history of medical implants who provided informed consent were eligible to participate in the study. Pregnant women were not eligible to participate in the study. Participants from the urban clinic were selected from a data set of an ongoing study, the HIV Hepatocellular Carcinoma in Uganda (H2U) study, which aims to determine the prevalence and progression of premalignant cirrhosis. The H2U study enrolled HIV-infected adults who provided informed consent to study procedures, including liver fibrosis measurement and laboratory testing. Pregnant women, persons with medical implants, and persons with a known history of liver disease were excluded from participation.

## Study Procedures

### Questionnaire

A structured interviewer-administered questionnaire was used for both studies to capture social-demographic information on age, gender, and self-reported use of alcohol, tobacco, and herbal medicines. For the participants in the rural clinic, clinical records were retrieved to inform on ART use, duration, and HIV viral load testing where available. In the event that these records were not captured in participants’ clinical records, we relied on self-reported information regarding information on when they started ART and how long they had been on therapy. ART-related information for the participants at the urban clinic was retrieved from their clinical records, including similar information of duration of use of ART and HIV viral load.

### Anthropometry

Height and weight measurements were taken for participants from both clinics. Height and weight were measured using a Seca Leicester stadiometer to the nearest 0.1 cm and a Seca 761 mechanical scale to the nearest 1 kg, respectively. Participants’ height and weight measurements were used to compute a body mass index (BMI) for each participant. BMI was calculated as weight (kg)/height (m)^2^. Participant categorization using BMI was as follows: BMI ≤ 18.5 “underweight,” BMI 18.5–24.9 “normal weight,” BMI 25–29.9 “overweight,” and BMI ≥ 30 “obese.”

### Blood Sample Processing

Participants from both clinics were bled under aseptic conditions for the purposes of serology (hepatitis B&C) and liver function testing. Blood samples for the participants from the rural clinic were transported within same day under suitable conditions to the Central Diagnostic Laboratory Services (CDLS) at the UVRI/MRC Uganda Research Unit Campus in Entebbe. The CDLS is an ISO-certified laboratory that processes varied volumes of samples from multiple ongoing research studies. Hepatitis B serology (HBV) testing was performed using the Hepanostika (HBsAg) ultra-confirmatory test (BioMérieux SA, Marcy l’Etoile, France). Hepatitis C (HCV) antibody testing was done using a fourth-generation enzyme-linked immunosorbent assay test (Innotest HCV Ab IV). Samples from the participants at the urban clinic were processed at the same premises as the clinic at the Infectious Diseases Institute in the Makerere University John Hopkins University (MUJHU) laboratory, a CAP-certified laboratory. Hepatitis B serology was performed using an enzyme immunoassay (Monolisa HBsAg Ultra 3; Bio-Rad). Hepatitis C antibody testing was done using third-generation enzyme immunoassay (Bio-rad Monolisa Anti-HCV PLUS).

### Transient Elastography

Liver stiffness measurements (LSMs) were taken using Fibroscan Echosens for participants at both clinics. At both clinics, the machine was operated by skilled personnel, taking 10 valid readings with an accuracy of 60% and interquartile range <30%. The median of these 10 readings was what was considered the final result for liver stiffness and was represented in kilo Pascals (KPa). The Fibroscan at the rural clinic only had an M-probe, whereas the Fibroscan at the urban clinic had both an M probe and an XL probe, the latter being appropriate for persons with a BMI ≥30.

## Data Analyses

The study data were stratified by clinic location because of the differences in the distribution of some risk factors for liver fibrosis, as shown by other studies done in-country [24, 25]. The data were also stratified by sex to control for effect modification based on prior evidence of similar studies in rural Uganda and to account for differences in choice of certain risky lifestyle habits dictated by gender [14, 26, 27]. The outcome liver fibrosis was assessed as a binary outcome, defined as LSM >7.1 KPa, a cutoff similar to those of prior studies for the purposes of comparison and also in correspondence to stage ≥F2 of METAVIR staging for liver fibrosis for most of the common causes of liver disease [14]. Proportions were used to describe sociodemographic, anthropometric, and clinical characteristics by clinic site. The prevalence of liver fibrosis was presented as a proportion for both study sites and compared using a chi-square test. The study population identified with liver fibrosis was stratified by clinic site and further described using proportions for sociodemography and clinical characteristics; comparison by clinic population was made using the chi-square test.

To determine factors associated with liver fibrosis, univariate analysis was conducted for each risk factor. Factors with a *P* value of ≤.15 were considered significant at this initial stage and used to build the multivariate model. Risk factors found to be insignificant in univariate analysis and yet known to cause liver fibrosis were retained in the multivariate model. Logistic regression was then conducted, and explanatory variables with a *P* value ≤.05 were considered significant. Analyses were performed using the STATA 12 statistical package.

## Patient Consent Statement

The study was conducted in accordance with the principles of the Declaration of Helsinki. Study approval for the rural-based study was given by the Uganda Virus Research Institute (GC/127/15/04/503 & GC/127/16/05/503), the School of Medicine Research Ethics Committee (SOMREC; #REC REF 2017–165), and the Uganda National Council of Science and Technology (UNCST; HS 1794 & ADM 154/212/01) research ethics committees. The H2U urban-based study was approved by the SOMREC (REF 2015–149), UNSCT (HS 1984), and the John Hopkins Medical Institutes Review Board (IRB 00086055). Written informed consent was obtained from all study participants for study participation, sample collection, and access to medical records.

## RESULTS

**Figure 2.**
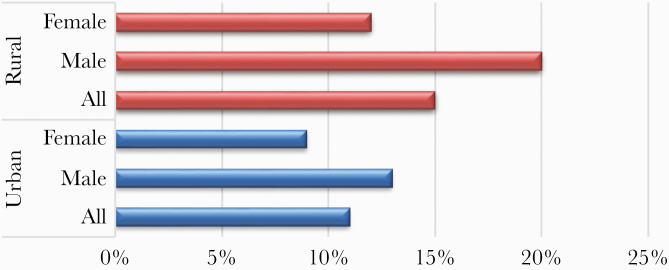
Prevalence of liver fibrosis among participants attending the Adult Infectious Diseases Clinic and General Population Clinic clinics between 2015 and 2017.

Table 1 shows the characteristics of the 2030 HIV-infected study participants attending the urban and rural clinics. The numbers of participants attending the urban and rural clinics were 1703 and 327, respectively. The majority (60%) of the study participants were female. The gender and mean age (urban population mean age, 44 years; rural population mean age, 45 years) were similar between the 2 clinics. The age distribution between the 2 clinics differed, with an older demographic attending the urban clinic. Participants from the urban clinic had been on ART for a longer duration than participants attending the GPC clinic and had better HIV viral load suppression rates (10 years vs 4 years; *P* = .001; 97% vs 77%; *P* < .001, respectively). Chronic HBV was nearly 3 times more prevalent among participants attending the urban clinic (11% vs 4%; *P* < .001), but there was no difference in the serological prevalence of HCV between participants attending both clinics. Participants in the urban clinic were more overweight and/or obese (*P* < .001). Participants attending the rural clinic had a higher proportion of having ever consumed alcohol (56% vs 43%; *P* < .001), tobacco (23% vs 16%; *P* = .002), and herbal medicine (27% vs 20%; *P* = .007). Liver fibrosis was significantly more prevalent among participants attending the rural clinic (15% vs 11%; *P* = .017) and even when stratified by gender, both male and female participants from the rural clinic had a higher prevalence of liver fibrosis than their counterparts in the urban clinic (Figure 2).

**Table 1.** Characteristics of HIV-Infected Patients Presenting at the AIDC and GPC Care Clinics in Urban and Rural Uganda (2015–2017)

Characteristic	Urban Site (n = 1703)	Rural Site (n = 327)	*P* Value
Male gender	678 (40)	125 (38)	.647
Age, y	44 ± 10.7	45 ± 10.9	.128
Age group			
<30 y	163 (10)	17 (5)	
30–39 y	311 (18)	76 (23)	
40–49 y	655 (38)	110 (34)	
50–59 y	441 (26)	88 (27)	
60–69 y	118 (7)	30 (9)	
70+ y	15 (1)	6 (2)	.008
Alcohol use	734 (43)	183 (56)	<.001
Tobacco use	266 (16)	74 (23)	.002
Herbal medicine use (n = 2015)	345 (20)	84 (27)	.007
Body mass index (n = 2024)			
Normal weight	1005 (59)	248 (76)	
Underweight	128 (8)	41 (13)	
Overweight	381 (22)	31 (10)	
Obese	184 (11)	6 (2)	<.001
Hepatitis B surface antigen test (n = 1757)			
Positive	195 (11)	12 (4)	<.001
Hepatitis C antibody test (n = 1784)			
Positive	28 (2)	5 (2)	.674
On ART (n = 2019)	1531 (90)	282 (89)	.722
ART duration (n = 1653), y	10 ± 3	4 ± 6	<.001
Detectable HIV RNA (≥1000 copies/mL; n = 1595)	41 (3)	52 (23)	<.001
Elevated liver transaminases			
Present	139 (9)	26 (8)	.385
Prevalence of liver fibrosis (n = 1956)	175 (11)	49 (15)	.017

Data are presented as No. (%) or mean ± SD.

Abbreviations: AIDC, Adult Infectious Diseases Clinic; ART, antiretroviral therapy; GPC, General Population Clinic.

The 224 study participants identified with liver fibrosis from both clinics were similar in gender and mean age distribution and proportion of ART coverage (Table 2). Participants from the rural clinic identified with liver fibrosis were more likely to have ever used tobacco (39% vs 21%; *P* = .009), have a shorter duration on ART (3 years vs 10 years; *P* < .001), and have detectable HIV viral loads (24% vs 4%; *P* < .001). On the other hand, participants at the urban clinic identified with liver fibrosis were more likely to be overweight and obese (*P* = .004) and had a 7 times higher prevalence of chronic HBV coinfection (14% vs 2%; *P* = .001). The prevalence of HCV and the proportional use of alcohol were similar in both populations from both clinics.

**Table 2.** Characteristics of 224 HIV-Infected Patients Attending the AIDC and GPC Care Clinics and Identified With Liver Fibrosis by Fibroscan (2015–2017)

Characteristic	Urban Clinic (n = 134)	Rural Clinic (n = 35)	*P* Value
Age, mean ± SD, y	46 ± 10.7	46 ± 11.1	.886
Gender (n = 223)			
Male	86 (49)	26 (53)	.653
Ever used of alcohol	78 (45)	27 (55)	.192
Ever used tobacco	36 (21)	19 (39)	.009
Ever used herbal medicines (n = 223)	39 (22)	13 (27)	.486
Body mass index^a^ (n = 222)			
Normal weight	103 (60)	34 (69)	
Underweight	12 (7)	9 (18)	
Overweight	43 (25)	5 (10)	
Obese	15 (9)	1 (2)	.007
Positive hepatitis B surface antigen test (n = 201)	25 (14)	1 (2)	.001
Positive hepatitis C antibody test (n = 206)	5 (3)	3 (6)	.353
On ART	157 (90)	41 (87)	.551
ART duration (n = 181), y	10.4 ± 3.3	3.7 ± 3.7	<.001
Detectable HIV viral load^b^ (n = 177)	6 (4)	9 (24)	<.001
Elevated liver transaminases^c^	27 (17)	3 (6)	.057

Data are presented as No. (%) or mean ± SD.

Abbreviations: AIDC, Adult Infectious Diseases Clinic; ART, antiretroviral therapy; GPC, General Population Clinic.

^a^World Health Organisation classification.

^b^≥1000 copies/mL.

^c^AIDS Clinical Trials Group Classification.

On assessing for the factors associated with liver fibrosis by gender and locality based on clinic location, we found history of tobacco use and serologic evidence of HCV to be common risk factors among men from both clinics (Table 3). Men from both clinics who had ever used tobacco had nearly twice the odds of presenting with liver fibrosis, and those who tested positive for HCV had 3 times the odds of having liver fibrosis (OR, 3.1; *P* = .028 urban participants; OR, 3.5; *P* = .028 rural participants). Risk factors unique to men attending the urban clinic were increasing age (OR, 1.3; *P* = .008), detectable HIV viral load (OR, 2.3; *P* = .002), and elevated liver transaminases (OR, 2.2; *P* = .002). Common factors associated with liver fibrosis among female participants from both clinics (Table 4) included elevated liver transaminases (OR, 2.3; *P* = .002 urban participants; OR, 2.4; *P* = .028 rural participants) and increasing age (OR, 1.3; *P* = .008 urban participants; OR, 1.3; *P* = .052 rural participants). History of tobacco use as a risk factor unique to female participants in the rural clinic was associated with 3 times the odds of having liver fibrosis.

**Table 3.** Factors Associated With Liver Fibrosis Among HIV-Infected Male Patients Attending AIDC and GPC Care Clinics in Urban and Rural Uganda (2015–2017)

	Urban Clinic		Rural Clinic	
Explanatory Variable	Adjusted Analysis	*P* Value	Adjusted Analysis	*P* Value
	OR (95% CI)		OR (95% CI)	
Age (10-y increase)	1.3 (1.1–1.6)	.008	1.0 (0.8–1.3)	.878
Ever used alcohol	0.6 (0.4–0.9)	.033	0.7 (0.4–1.2)	.193
Ever used tobacco	1.7 (1.1–2.7)	.022	1.7 (1.0–2.7)	.035
Ever used herbal medicine	1.1 (0.7–1.7)	.754	1.1 (0.6–2.0)	.746
Hepatitis B surface antigen test positive	1.1 (0.6–1.9)	.728	1.4 (0.7–2.5)	.309
Hepatitis C antibody positive	3.1 (1.1–8.6)	.028	3.5 (1.1–11.0)	.030
On ART	0.8 (0.9–7.2)	.840	0.9 (0.1–8.5)	.936
Detectable HIV viral load (≥1000 copies/mL)	2.3 (1.2–4.6)	.002	1.8 (0.9–3.7)	.101
Elevated liver transaminases^a^	2.2 (1.3–3.7)	.002	1.4 (0.7–2.5)	.314

Abbreviations: AIDC, Adult Infectious Diseases Clinic; ART, antiretroviral therapy; GPC, General Population Clinic; OR, odds ratio.

^a^AIDS Clinical Trials Group classification.

**Table 4.** Factors Associated With Liver Fibrosis Among HIV-Infected Females Patients Attending the AIDC and GPC Care Clinics in Urban and Rural Uganda (2015–2017)

	Urban Clinic		Rural Clinic	
Explanatory variable	Adjusted Analysis	*P* Value	Adjusted Analysis	*P* Value
	OR (95% CI)		OR (95% CI)	
Age (10-y increase)	1.3 (1.1–1.6)	.008	1.3 (1.0–1.6)	.052
Ever used alcohol	0.6 (0.4–0.9)	.016	0.6 (0.4–1.1)	.082
Ever used tobacco	1.5 (0.9–2.5)	.091	2.9 (1.5–5.9)	.003
Ever used herbal medicine	1.2 (0.7–1.9)	.474	1.0 (0.6–1.8)	.949
Hepatitis B surface antigen positive	1.1 (0.7–2.0)	.612	0.4 (0.1–1.1)	.072
Hepatitis C antibody positive	1.9 (0.6–6.0)	.263	2.2 (0.6–9.0)	.259
On ART	-	-	0.6 (0.1–4.9)	.594
Detectable HIV viral load (≥1000 copies/mL)	1.4 (0.6–3.3)	.403	1.4 (0.7–2.9)	.404
Elevated liver transaminases^a^	2.3 (1.3–3.9)	.002	2.4 (1.1–5.3)	.028

Abbreviations: AIDC, Adult Infectious Diseases Clinic; ART, antiretroviral therapy; GPC, General Population Clinic; OR, odds ratio.

^a^AIDS Clinical Trials Group classification.

## DISCUSSION

The prevalence of liver fibrosis among HIV-infected patients attending care clinics both in urban and rural Uganda was high, and significantly higher among those attending the rural care clinic. The prevalence of liver fibrosis among HIV-infected persons attending the rural care clinic was similar to that reported in a previous study (15% vs 17%) conducted at a time when ART access was limited [14]. Studies done elsewhere within SSA have reported a similar range in prevalence of liver fibrosis among HIV-infected persons ranging between 2% and 24% depending on the study population and technique used [15–17, 28, 29]. Among Western populations, the prevalence of liver fibrosis among HIV-infected patients was similar to the findings of our study and other studies in SSA, with estimates of 16%–29% [20]. Antiretroviral therapy has been demonstrated to reduce risk of liver fibrosis via adequate viral suppression [8, 30]. In resource-limited settings, rollout of HIV care programs started in urban settings long before their initiation within hard-to-reach areas [23]. Timing of rollout impacts the ART experience and consequently infection control. In this study, the mean duration of ART was longer among the participants in the urban clinic (10 years vs 4 years), and a higher proportion had controlled HIV infection. Differences in these HIV-related factors may play a significant role in the observed difference of the burden of liver fibrosis observed between these 2 populations.

Tobacco use was a common risk factor regardless of locality or gender. Tobacco is a carcinogen that increases risk for HCC and has been documented to compromise the cancer surveillance system of the body [31]. Other literature has also demonstrated smoking to be an independent risk factor for liver fibrosis [32, 33]. Among men from both localities, HCV was identified as a common risk factor for liver fibrosis. The transmission routes for HCV are not fully understood within SSA; however, established transmission routes from other studied populations are mainly associated with high-risk behavior common among men [34]. This may explain why HCV was a risk factor among men and not women in this study, although we did not assess for risk behavior such as risky sexual behavior, intravenous drug abuse, or other practices like tattooing. Chronic HBV was not associated with severe liver disease among the populations in either clinic. The current first-line ART in Uganda includes 2 drugs with activity against HBV, tenofovir and lamuvidine, the combination of which reduces the chances of drug-resistant HBV infection. Elevated liver transaminases, although shown to be an associated factor of liver fibrosis, may be a marker of disease severity, as demonstrated by other studies [12, 35–37].

A considerable proportion of our study participants were using herbal medicines, but we did not identify herbal medicines as a risk factor. Previous studies conducted in rural Uganda found an association between liver fibrosis and consumption of herbal medicines [38]. Given that the earlier studies were conducted in a time of relatively limited ART access, it is probable that HIV-infected persons resorted to alternate remedies of therapy. It is also probable that they consumed them in large quantities and for long durations, posing a risk of liver injury. Presently there is a concerted effort underway to regulate the production of herbal medicines in the country to ensure their safety for human use [39, 40]. Some herbal medicines possess antifibrotic properties, although there remains limited knowledge of the whole scope of their action [41]. Although alcohol abuse is a well-documented risk factor for liver disease, we on the contrary found it to be a protective factor only among the participants in the urban clinic. Given the well-developed and consistent counseling services given to patients at the urban clinic (including substance abuse), it may be possible that there exists a countercompensatory behavior adopted by these persons and not necessarily the effect of alcohol that could not be accounted for by our study. We also acknowledge that it is possible that alcohol intake was underreported, because most persons will provide socially acceptable responses.

We acknowledge that this study had several limitations. We also did not assess for other possible explanatory variables for the proportion of liver fibrosis we observed, such as presence of schistosomiasis, which is very common in this setting, and co-administered drugs that are toxic to the liver such as antituberculous drugs. This study does not account for the prior exposures over the life course of the participants. Thus we did not take into account the impact of those early exposures like migration history on the impact of the development of liver fibrosis. The urban study population was from a tertiary care unit and may not be very representative of the wider population of HIV-infected persons in urban Uganda. We did not assess for HBV viral load and used a serological marker for HCV, which is not necessarily a marker for active infection and has a high rate of false positives, due to financial constraints. We acknowledge the limitation in assessing some variables as binary, such as the use of alcohol, which may have affected the estimates observed. Participants were not asked to fast before conducting liver stiffness measurements, and this may have impacted some of the results observed. This study, however, using a readily acceptable noninvasive technique in the screening of liver fibrosis, provides evidence of a high burden of liver fibrosis among HIV-infected persons in a resource-limited setting, demonstrates differences in disease distribution by locality, and may provide further evidence for more evaluation on comorbid conditions as risk factors of liver fibrosis other than opportunistic infections in the era of ART. Our characterization of the risk profile for liver fibrosis provides valuable information that may can inform future intervention trials and formulate strategies for primary prevention.

## CONCLUSIONS

Liver fibrosis is a common condition among HIV-infected persons in Uganda in the era of ART. Persons at risk are those with poorly controlled HIV infection, coinfected with viral hepatitis C, using tobacco products, and having elevated liver transaminases. We recommend that the keys to reducing risk of liver fibrosis and/or liver cancer among HIV-infected persons in Uganda may be the use of ART drugs with efficacy to achieve HIV viral suppression. We also recommend that the current national tobacco bill in Uganda be enforced and strengthened to control for the access and use of noncommercialized tobacco products. More studies with confirmation of hepatitis C, as well as treatment of those infected, are recommended to prevent liver fibrosis and its complications.
